# Differential Metabolomic Signatures in Boar Sperm with Varying Liquid Preservation Capacities at 17 °C

**DOI:** 10.3390/ani15152163

**Published:** 2025-07-22

**Authors:** Serge L. Kameni, Notsile H. Dlamini, Jean M. Feugang

**Affiliations:** Department of Animal and Dairy Sciences, Mississippi State University, Starkville, MS 39762, USA; sk2086@msstate.edu (S.L.K.); nhd30@msstate.edu (N.H.D.)

**Keywords:** chilled storage, metabolites, metabolome, motility, pig, sperm

## Abstract

In the swine industry, artificial insemination (AI) primarily uses chill-stored semen, making sperm preservation crucial for reproductive success. However, sperm quality declines at varying rates during chilled storage at 17 °C, distinguishing high-survival semen from low-survival semen. This study investigates the metabolomic profiles of boar sperm with different abilities to survive liquid storage. We analyzed sperm motility, kinematics, and morphology in freshly extended (Day 0) and 7-day stored AI semen doses. The AI semen doses were classified as high-motile (HM) or low-motile (LM) based on sperm motility after 7 days of storage (Day 7). Metabolomic data were collected in positive (ESI+) and negative (ESI−) ion modes using a Vanquish Flex UPLC coupled with a Q Extractive Plus. We consistently detected 442 metabolites (251 in ESI+, 167 in ESI−, and 24 in both) across samples and storage durations. In freshly extended and 7-day stored AI doses, we identified 42 and 56 differentially expressed metabolites (DEMs), respectively. A clustering analysis showed significant changes in DEMs between the HM and LM samples. These DEMs were mainly enriched in amino acid metabolism, the pentose phosphate pathway, glycerolipid metabolism, glyoxylate and dicarboxylate metabolism, terpenoid backbone biosynthesis, etc. In summary, this study highlights the metabolomic differences between semen doses with varying abilities to survive liquid storage. Glyceric acid and lysoPC(20:3) emerged as potential markers for sperm preservation.

## Simple Summary

In modern pig farming, reproduction is predominantly achieved through artificial insemination, which consists of inserting spermatozoa into the female reproductive tract. The success of this technique relies on several factors, especially the quality of the sperm used. Following collection, fresh ejaculates are processed into seminal doses that can be stored for days. However, during storage, the sperm quality declines at different rates among ejaculates, leading to inconsistent fertility outcomes. To identify those with varying storage tolerances early and thus improve reproductive outcomes, we investigated the metabolome—the complete set of small molecules that mirror cell activity—of spermatozoa with differential abilities to withstand storage. The findings revealed that the spermatozoa possess distinct metabolome profiles, which could facilitate early diagnosis upon semen collection. Specifically, the prevalence of two metabolites, glyceric acid and lysoPC(20:3), emerges as a practical indicator for the timely characterization of semen with differential abilities to sustain storage. These insights have the potential to improve semen dose management, boosting reproductive efficiency and profitability.

## 1. Introduction

Semen preservation constitutes a core step in the sustainability of swine breeding, ensuring acceptable sperm quality for improving the utilization efficiency of ejaculates [1]. Sperm preservation consists of reducing cellular metabolism while prolonging the fertile life of spermatozoa. Precisely, optimal sperm preservation may maintain an adequate energy balance to enable the spermatozoa to complete fertilization-related functions [2,3]. This is particularly relevant as the spermatozoa energy expense and, thus, the sperm function is at risk during prolonged storage [4]. Numerous factors, such as animal management, nutrition, environment, age, and genetics influence boar reproductive performance [5,6] and, consequently, sperm preservability, which further depends on semen handling protocols, hygienic conditions, storage temperature, and extenders’ composition [2]. However, despite the constant optimization of semen handling protocols, notably through the development of highly efficient extenders and the adjustment of the preservation temperature to 4–5 °C to prevent bacterial growth and allow antibiotic-free storage, the gradual decline in sperm quality with prolonged storage remains a hallmark of the process [7,8], limiting the widespread use of preserved semen in artificial insemination (AI). In AI programs, threshold values are set for sperm concentration, viability, motility, and morphology to ensure only ejaculates of a high caliber are processed to produce seminal doses with high fertility and prolificacy potential. Interestingly, growing evidence shows that boar ejaculates qualified to be processed in AI doses respond differentially to storage [9,10,11]. Indeed, various aspects of boar management and semen handling significantly influence the quality of boar semen, exhibiting a sire-dependent variability that affects the ability of semen to endure preservation and subsequently support successful conception rates [12]. To optimize the productivity of boar studs, it is necessary to understand the dynamics sustaining the differential ability of ejaculates to withstand preservation.

Omics technology provides the opportunity to explore the intricate molecular mechanisms that govern cellular function. Coupled with bioinformatic tools, omics, particularly proteomics and transcriptomics, are routinely used to analyze the molecular content of spermatozoa and biofluids to identify sperm preservation and fertility biomarkers [9,13,14,15,16]. Moreover, some of these biomarkers varied according to the season, potentially indicating the impact of heat stress, ambient temperature, and photoperiod on sperm function [17,18]. Metabolomics, another branch of functional genomics, is widely regarded as the omics discipline that most closely reflects the phenotype [19], allowing the exploration of end-products that result from biochemical reactions and related pathways. As such, the sperm metabolome may constitute an important niche of data for deciphering biomarkers associated with the sperm phenotype. In boars, it has been shown that greater levels of glycolysis-derived metabolites, assessed by spermatozoa, depict high-quality spermatozoa resulting in higher percentages of embryos following in vitro fertilization [20]. Moreover, chilled and post-thaw boar sperm quality has been associated with the metabolomic signatures of the spermatozoa [21,22] and seminal plasma [23,24]. The differently abundant metabolites reported in these studies were primarily amino acids, lipids, and peptides [21,22,23,24]. These metabolites are involved in pathways related to energy production, amino acid, and fatty acid biosynthesis. Furthermore, sperm-borne L-citrulline and seminal plasma-derived tryptophan have been proposed as potential sperm freezability markers in boars [25]. In sperm cells, the different lipid metabolism levels and long-chain polyunsaturated fatty acids in the plasma membrane are considered key factors contributing to differences in sperm freezability [22]. Hence, spermatozoa from ejaculates with contrasting sperm attributes possess distinct metabolic profiles, indicating the key role of metabolites in ensuring sperm quality.

Therefore, the present study aims to identify the metabolomic biomarkers associated with boar sperm liquid preservation ability and to analyze the dynamics of the sperm metabolome over storage. A detailed analysis of the metabolites related to sperm preservation may improve strategies for boar semen storage and predict sperm survival. This advancement aims to enhance elite male selection, address production challenges, and support the sustainable development of the swine breeding industry.

## 2. Materials and Methods

### 2.1. Ethics Statement

The semen samples used in this study were surplus from routine production at a commercial boar stud facility and were not collected explicitly for research. Their collection and handling followed standard industry protocols, ensuring ethical and humane treatment of the animals. These samples were exempt from ethical clearance as byproducts of typical production activities.

### 2.2. Semen Collection and Processing

Boars were raised at a commercial stud facility (Prestage Farms, West Point, MS, USA), kept in a ventilation-controlled environment, and provided with a concentrated diet tailored to adult boars’ needs, alongside unlimited water access. Ejaculates were obtained from healthy adult Duroc boars (1–2.5 years old) using the gloved-hand method and only ejaculates with total motility ≥70% and normal morphology ≥ 80% were further processed, according to standard procedures. Raw semen was extended with NUTRIXcell+ extender (IMV Biotechnologies; Brooklyn Park, MN, USA) and packaged in doses of 45 to 50 mL within transparent semen storage bags. Freshly extended semen doses were immediately transported to the laboratory within 30 min for analysis in an insulated polystyrene box containing refrigerant gel packs. Upon arrival at the laboratory, each semen dose was divided into two aliquots for analyses on Day 0 (collection day) and Day 7. Samples were cooled at 17 °C for up to 7 days (Day 7), with gentle shaking every 12 h to prevent sperm sedimentation.

### 2.3. Assessment of Sperm Parameters and Classification of Semen Doses

The motion and morphological characteristics of semen doses were assessed as previously described [10,16], using aliquots of samples incubated for 15 min in a 37 °C water bath. Subsequently, 2 µL of the incubated aliquot was loaded into a disposable counting chamber for analysis using a computer-assisted sperm analyzer (CEROS II, IMV Biotechnologies, Brooklyn Park, MN, USA). The system operated at 60 Hz (60 frames/sec) with settings for head size ranging from 10 to 50 μm and a minimum tail brightness of 86. Motile sperm were defined by an average path velocity (VAP) > 45 μm/s and a straight-line velocity (VSL) > 5 μm/s. Progressive sperm were characterized by a VAP > 45 μm/s and straightness > 45%. Samples were evaluated for total motility (MOT), progressivity (PM), curvilinear velocity (VCL), average path velocity (VAP), and straight-line velocity (VSL). Additionally, the normal morphology and morphological defects (distal droplets, proximal droplets, bent tails, and coiled tails) were assessed using the CASA morphology module. All the samples were evaluated by the same observer, and for each analysis, a total of 250 to 300 spermatozoa were assessed.

Extended single-sire semen doses from four to five individual boars were processed on six independent occasions, culminating in the analysis of a total of 26 semen doses (1 per boar) at both fresh collection (Day 0) and after chilled storage (Day 7) (Table 1).

**Figure 1:**
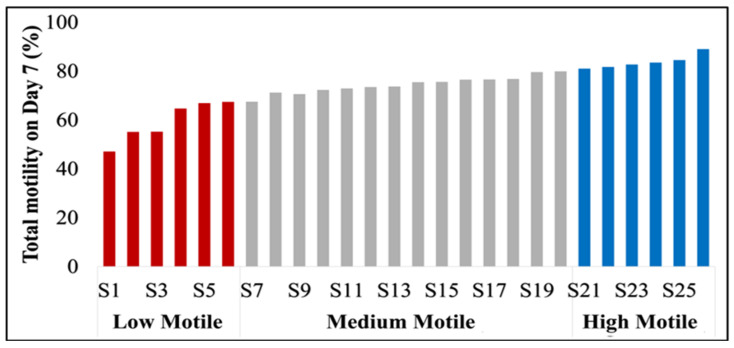
Ranking of boar semen samples based on sperm motility on Day 7 post-storage. A total of 26 single-sire boar semen samples or doses (S1 to S26) were evaluated on Day 7, and semen were classified as low (LM), medium (MM), or high motile (HM).

The semen samples were classified based on motility as follows: low motile (LM: <70%), medium motile (MM: ≥70% and <80%), and high motile (HM: ≥80%), using the motility percentages observed on Day 7 after analyzing all 26 samples (Figure 1). The LM and HM groups from both Day 0 and Day 7 were chosen for subsequent metabolomic analyses.

For the metabolomic analysis, only extended semen samples from the high-motility (HM, *n* = 6) and low-motility (LM, *n* = 6) groups were included. These samples underwent centrifugation at 800× *g* for 5 min at 4 °C, following sperm motility assessments conducted on both Day 0 and Day 7 of storage. The resulting pellets were washed twice with phosphate-buffered saline. The final sperm pellets, each containing approximately 100 × 10^6^ spermatozoa, were rapidly stored at −80 °C for further analysis of the LM and HM groups, as identified on Day 7 and traced back to Day 0.

### 2.4. Mass Spectrometric Analysis

#### 2.4.1. Metabolite Extraction

Sperm pellets of frozen LM and HM spermatozoa at Day 0 (LMD0/HMD0) and Day 7 (LMD7/HMD7) were thawed on ice, and 300 μL of 80% methanol was added. The mixture was vortexed for 30 s and then sonicated in an ice-water bath for 30 min. Subsequently, the samples were kept at −20 °C for 1 h, vortexed again for 30 s, and centrifuged (12,000 rpm, 10 min, 4 °C). After centrifugation, 200 μL of the supernatant was transferred into a vial, and 5 μL of DL-o-chlorophenylalanine (0.14 mg/mL) was added as an internal standard. The mixture was finally filtered through a 0.22 μm filter for LC-MS analysis. Equal volumes of extract from each sample were combined to obtain quality control samples.

#### 2.4.2. UPLC/MS Analysis

Metabolomic data were acquired in positive and negative ion modes using a Vanquish Flex UPLC coupled with a Q Extractive Plus (Thermo Fisher Scientific; Waltham, MA, USA) equipped with a heated ESI source. A Waters T3 column (100 mm × 2.1 mm × 1.8 μm; Waters, Milford, MA, USA) operated at 40 °C was used for liquid chromatography separation. The mobile phases consisted of two solvents, A (water with 0.05% formic acid) and B (acetonitrile). This mobile phase system was run in a gradient elution as follows: 5% B at 0–1 min; 5–95% B at 1–12.5 min; 95% B at 12.5–13.5 min; 95–5% B at 13.5–13.6 min; 5% B at 13.6–16 min. The flow rate of the mobile phase was 0.3 mL/min. The sample manager temperature was maintained at 4 °C. Mass spectrometry parameters in positive (ESI+) and negative (ESI−) modes were set as follows: ESI+: heater temperature 300 °C; sheath gas flow rate, 45 arb; aux gas flow rate, 15 arb; sweep gas flow rate, 1 arb; spray voltage, 3.0 kV; capillary temperature, 350 °C; S-Lens RF level, 30%. ESI−: heater temperature 300 °C, sheath gas flow rate, 45 arb; aux gas flow rate, 15 arb; sweep gas flow rate, 1 arb; spray voltage set to 3.0 kV (ESI+) and 3.2 kV (ESI−); capillary temperature, 350 °C; S-Lens RF Level adjusted to 30% (ESI+) and 60% (ESI−).

### 2.5. Data Analysis

Sperm quality data were analyzed with SPSS (Version 29.0.1.0 (171), SPSS Inc., Chicago, IL, USA). Data normality (Shapiro–Wilk test) was verified, and the Student *t*-tests were used to compare samples according to semen motility and storage durations, with significance set at *p* < 0.05. Results are presented as mean ± standard error of the mean (SEM).

For metabolomic analysis, raw data were acquired and aligned using the Compound Discoverer (3.0, Thermo) based on the mass-to-charge ratio (*m*/*z*) values and the retention time of the ion signals. Metabolites from both ESI− and ESI+ were merged and imported into the SIMCA-P program (version 14.1, Umetrics, San Jose, CA, USA) for multivariate analysis. Supervised regression modeling was then performed on the dataset using orthogonal partial least squares discriminant analysis (OPLS-DA) to identify the difference between groups and the major metabolites associated. The candidate metabolite biomarkers were filtered and confirmed by combining the results of the variable importance in projection or VIP values (VIP > 1.5) and paired Student’s *t*-test (*p* < 0.05). VIP values were used to identify the most influential metabolites characterizing the samples according to phenotypes (HM or LM) in freshly extended (Day 0) and 7-day stored samples (Day 7).

The chemical structures of metabolites were identified according to the Human Metabolome Database (www.hmdb.ca; accessed on 30 August 2024) using data of accurate masses and MS/MS fragments. When necessary, further confirmation was acquired through comparisons with authentic standards, including retention times and MS/MS fragmentation patterns.

Mean values of metabolite contents from biological replicates of both HM and LM groups were used to calculate the metabolite ratio. After the logarithmic transformation of the data, median-centered ratios were subsequently normalized. Hierarchical clustering analysis (HCA) was performed using the complete linkage algorithm of the program Cluster 3.0 (Stanford University), and the results were visualized using Pheatmap 1.0.12 (Raivo Kolde). Metabolite ratios from two independent experiments involving significant metabolites were used for HCA, and those with VIP ≥ 1.5 and *p* < 0.05 were further classified as potential biomarkers. The Kyoto Encyclopedia for Genes and Genomes (KEGG; www.kegg.jp; accessed on 30 August 2024) pathway database and MetaboAnalyst (www.metaboanalyst.ca; accessed on 30 August 2024) were used to explore the underlying metabolic pathways. Lastly, the metabolites were grouped into clusters according to their expression profiles during storage, using the Mfuzz Bioconductor package [26]. Metabolic pathways with *p*-values < 0.05 were considered significantly enriched.

## 3. Results

### 3.1. Identification and Characterization of High- and Low-Motile Semen

**Figure 2:**
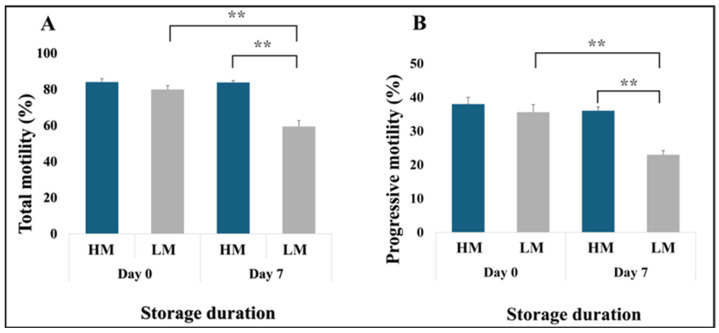
Sperm motility of high- (HM) vs. low-motile (LM) freshly extended (Day 0) and 7-day stored (Day 7) boar semen. (**A**) Total motility and (**B**) progressive motility. *N* = 6 for each group; ** *p* ≤ 0.01.

**Figure 3:**
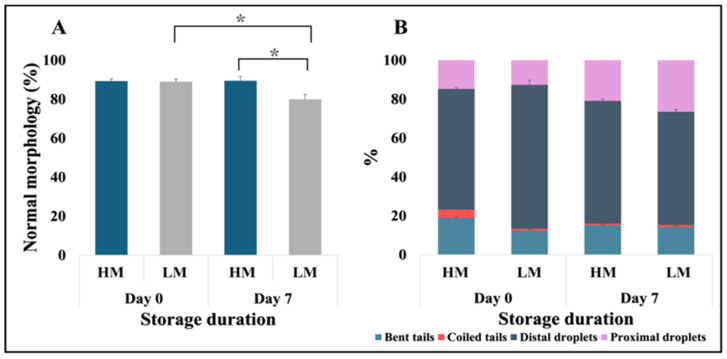
Sperm morphology of high-(HM) vs. low-motile (LM) freshly extended (Day 0) and 7-day stored (Day 7) boar semen. (**A**) Normal morphology. (**B**) Sperm defects. *N* = 6 for each group; * *p* < 0.05.

**Figure 4:**
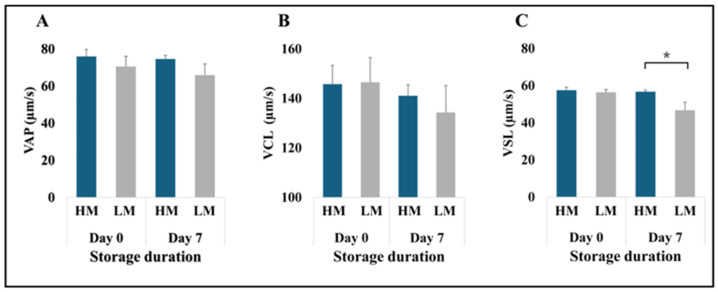
Sperm velocities of freshly extended (Day 0) and 7-day stored (Day 7) boar semen. (**A**) Average path velocity (VAP); (**B**) curvilinear velocity (VCL); (C) straight-line velocity (VSL). *N* = 6 for each group; * *p* < 0.05.

Table 1 shows sperm motility and morphology data, organized by experimental replicates, which led to the classification of semen doses into low-motile, medium-motile, and high-motile groups. The study specifically focused on low- and high-motile semen for metabolomic studies. The effects of 7-day chilled storage on sperm motility (total and progressive), morphological traits, and velocities are summarized in Figure 2, Figure 3 and Figure 4, respectively. Although HM and LM samples were comparable on Day 0 of storage (*p* = 0.09), significant differences in total motility (Figure 2A) and progressivity (Figure 2B) were observed on Day 7 of storage (*p* < 0.01), which led to categorizing AI doses into low- or high-motile semen groups in response to chilled storage.

Furthermore, sperm attributes in the low-motile semen (LM) group exhibited a significant decline over the storage period, with motility dropping notably (*p* < 0.01) from Day 0 (MOT = 79.92 ± 2.18%, PM = 35.63 ± 2.27%) to Day 7 (MOT = 59.40 ± 3.35%, PM = 23.00 ± 1.20%). In contrast, the high-motile semen (HM) group demonstrated consistent motility levels throughout the same timeframe. Specifically, the motility in the HM group was comparable between Day 0 (MOT = 84.02 ± 1.84%, PM = 38.02 ± 2.01%) and Day 7 (MOT = 83.73 ± 1.17%, PM = 36.07 ± 1.11%), indicating a robust preservation of sperm motility over time. Notably, the percentage of spermatozoa exhibiting normal morphology reflected the trends observed in motility (Figure 3A), with distal droplets identified as the major contributor to sperm abnormalities (Figure 3B). Interestingly, the proportion of spermatozoa with a coiled tail was four times higher in the HM group on Day 0 than in the other groups.

In contrast, except for the VSL on Day 7 (Figure 4C), no significant differences in sperm velocities were observed between groups or across storage durations (*p* > 0.05, Figure 4A,B).

### 3.2. Total and Differentially Expressed Metabolites (DEMs) in Spermatozoa Between Low- and High-Motile Semen

**Figure 5:**
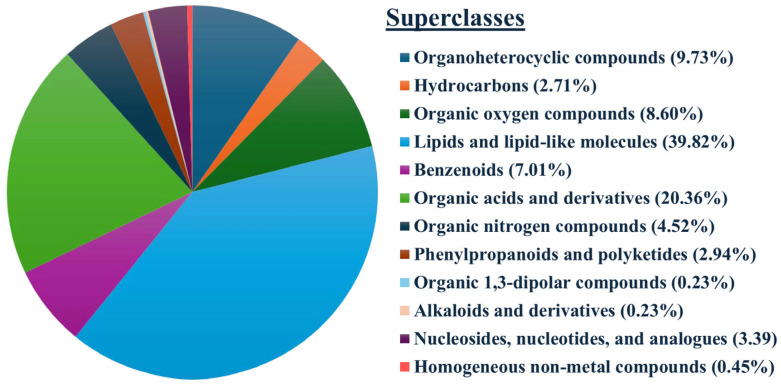
Distribution of boar sperm metabolites in chemical classes.

The metabolites were identified and categorized based on their chemical classes. The major classes included lipids and lipid-like molecules, organic acids and derivatives, organoheterocyclic compounds, organic oxygen compounds, and benzenoids, accounting for approximately 85.5% of the total metabolites detected (Figure 5).

**Figure 6:**
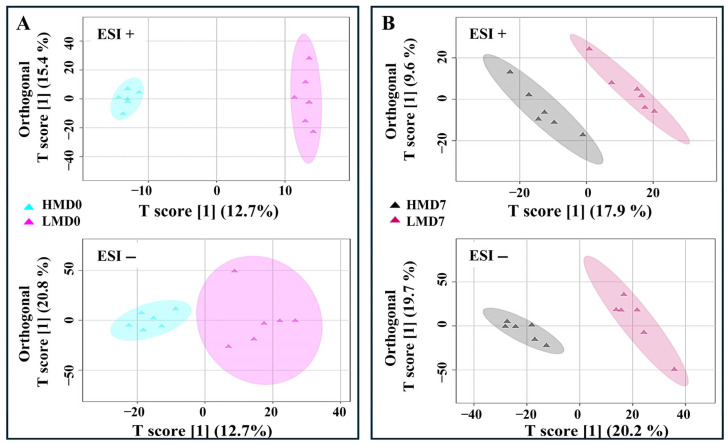
OPLS-DA score plots showing the separation between high-motile (HM) and low-motile (LM) boar spermatozoa on Day 0 (**A**) and Day 7 (**B**) of storage based on metabolome. Data were acquired in positive (ESI+) and negative (ESI−) ion modes.

To appraise the difference in the boar spermatozoa metabolomic profile between low- and high-motile semen and identify key metabolites, the OPLS-DA was performed using the 442 metabolites (251 with ESI+; 167 with ESI−; and 24 shared between ESI+ and ESI−) consistently detected across samples and storage duration. The details of all the detected metabolites are provided in Supplementary File S1. The OPLS-DA score scatter plots showed a clear separation between the groups in the positive and negative modes for both Day 0 and Day 7 of storage (Figure 6A,B). The VIP derived from the OPLS-DA analysis was used to identify the key metabolites driving the separation between the low- and high-motile AI doses.

**Figure 7:**
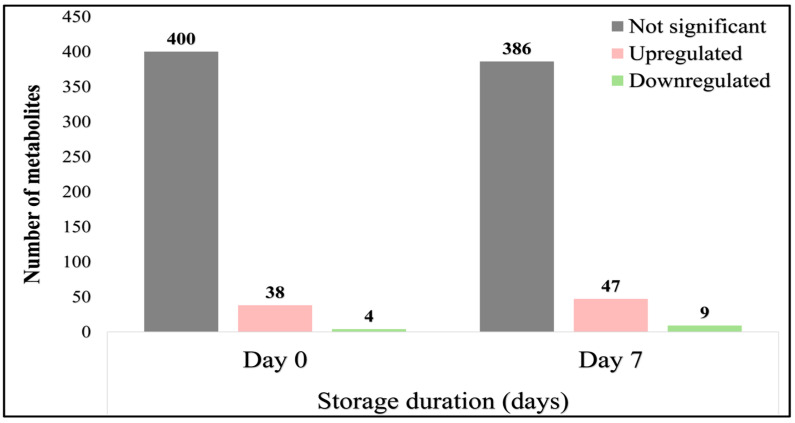
Distribution of differentially expressed metabolites between low- and high-motile semen in freshly extended (Day 0) and 7-day stored (Day 7) samples.

Data filtering (VIP score > 1.5 and *p* < 0.05) applied to the 442 unique metabolites identified through both negative (ESI−) and positive (ESI+) ion modes revealed significant dysregulation in 42 (~9.5%) and 56 (~12.7%) of the metabolites (referred to as differentially expressed metabolites or DEMs) between low- and high-motile semen on Days 0 and 7 of storage, respectively (Supplementary File S1). On Day 0, this analysis identified 38 upregulated and 4 downregulated DEMs in the HM group, while on Day 7, there were 47 upregulated and 9 downregulated DEMs (Figure 7).

Table 2 presents the predominant or most influential DEMs (VIP score > 2 and *p* < 0.05) detected on both days of storage. This list includes notable DEMs such as carvotanacetone, arachidoyl ethanolamide, palmitoleoylethanolamide, emulphor, and 6-hydroxyshogaol in ESI+ and N-dodecylsarcosinate, 5-diphosphomevalonic acid, 2-hydroxystearic acid, PG(a-13:0/a-13:0), glyceric acid, and LysoPE(20:4) in ESI−.

### 3.3. Cluster Analysis of Differentially Expressed Metabolites and Identification of Potential Biomarkers of Low- and High-Motile Semen Phenotypes

**Figure 8:**
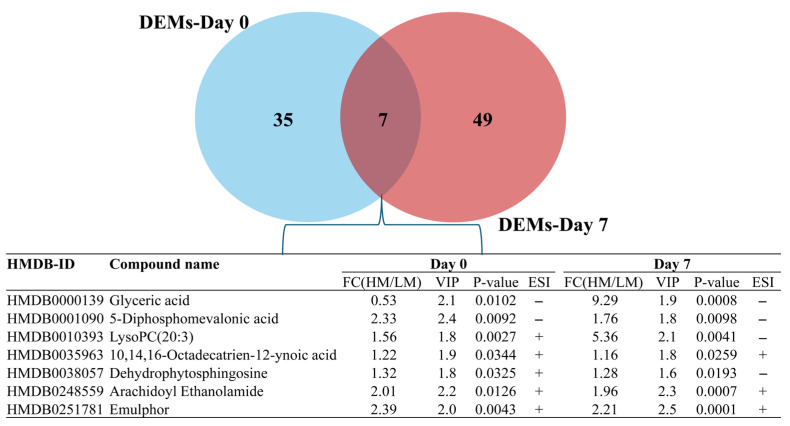
Selected differentially expressed metabolites candidate biomarkers detected in both freshly extended (Day 0) and 7-day stored (Day 7) boar semen.

**Figure 9:**
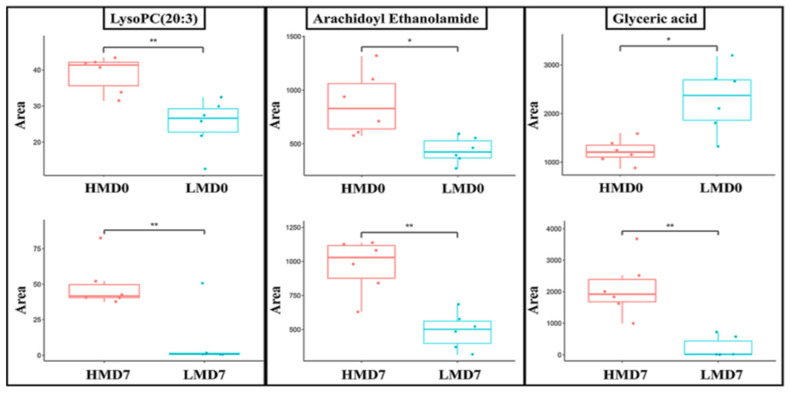
Boxplots of selected differentially expressed metabolites between high- and low-motile semen common to Day 0 (**Upper** panel) and Day 7 (**Lower** panel) of storage. HMD0 and HMD7: high-motile semen on Day 0 and Day 7; LMD0 and LMD7: low-motile semen on Day 0 and Day 7. * *p* < 0.05 and ** *p* ≤ 0.01.

Hierarchical clustering of the DEMs was implemented to assess the expression pattern of each metabolite in the spermatozoa between the LM and HM groups. The corresponding heatmaps are provided in Supplementary File S1. Notably, the differential expression patterns of metabolites were more pronounced in the ESI+ mode than in the ESI− mode on Day 0. Both ion modes exhibited distinguishable expression patterns of DEMs on Day 7. Figure 8 and Figure 9 illustrate the DEMs simultaneously detected in freshly extended (Day 0) and 7-day stored (Day 7) semen samples, which could serve as candidate biomarkers to differentiate LM from HM. These candidates include glyceric acid, arachidoyl ethanolamide, and lysoPC(20:3), which showed significantly increased fold-change expressions in the HM group during storage. Interestingly, these three candidates appear among the most influential metabolites during storage (VIP ≥ 2.0 and *p* < 0.05; Table 1). The list and details of other candidates are provided in Supplementary File S1.

**Figure 10:**
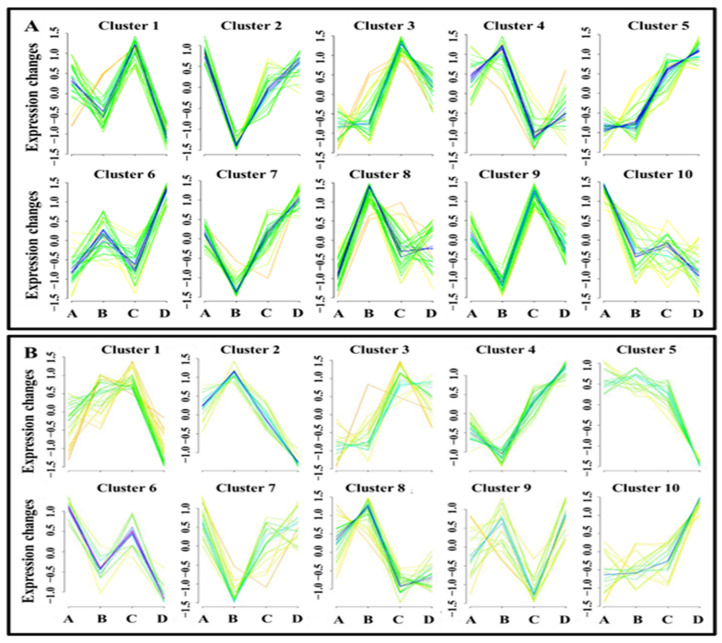
Mfuzz clusters of metabolites detected in the positive (**A**) and negative (**B**) ion modes. The clusters illustrate metabolite expression patterns between low- and high-motile spermatozoa on Day 0 and Day 7 of storage. Each individual line depicts the profile of a metabolite across groups and storage durations. Different colors depict different degrees of membership in the cluster. Violet, dark blue, light blue—high degree of membership; green—medium degree of membership; and yellow—poor degree of membership. Semen groups are represented by **A** for HMD0, **B** for LMD0, **C** HMD7, and **D** for LMD7.

Furthermore, the metabolites detected on both days were grouped into clusters (10 for each ion mode) using the Mfuzz method (Figure 10A,B). The analysis revealed various sperm metabolomic clusters, with many exhibiting distinct abundance patterns across sample groups. Notably, five clusters exhibited high metabolomic abundance in the HM group on Day 0 (Clusters 2 and 10 in ESI+) and Day 7 (Clusters 1, 3, and 9 in ESI+). Similarly, five clusters demonstrated a high abundance of metabolites in the LM group on Day 0 (Clusters 4 in ESI+, 2 and 8 in ESI−) and Day 7 (Clusters 6 in ESI+ and 10 in ESI−). In detail, the expression patterns of Cluster 10 in ESI+—with adenosine (VIP = 2.0, *p* = 0.009) as a core member—and Cluster 3 in ESI+—with dodecatrienoic acid (VIP = 2.2, *p* = 0.001) as a core member—were significantly associated with HM spermatozoa on Day 0 and Day 7, respectively. Moreover, Cluster 9 in ESI+ and Cluster 6 in ESI−, with octadecanamide (VIP = 1.55, *p* = 0.023) and lactic acid (VIP = 1.62 and *p* = 0.018) as core members, respectively, displayed elevated expression levels in HM spermatozoa on both days. While Cluster 4 in ESI+ and Clusters 2 and 8 in ESI− with glutamyltyrosine, uric acid, and hypotaurine as core members, respectively, showed high metabolic expression in LM spermatozoa on Day 0, only Cluster 10 in ESI− with monoethylexyl phthalic acid as core member depicted the same pattern in LM on Day 7. In terms of storage sustainability, Cluster 5 in ESI− with lysoPE(22:6), lysoPC(22:6), and 2-hydroxystearic acid as core members exhibited elevated expression patterns in freshly extended semen (Day 0). Conversely, Cluster 5 in ESI+ and Cluster 3 in ESI−, which were strongly associated with hexadecasphingosine and arachidonic acid (ESI−), respectively, presented a pattern of gradually increasing abundance from Day 0 to Day 7 (Figure 10A,B). More detailed information on the key metabolites associated with sperm motility status (HM and/or LM) and their storage resilience can be found in Supplementary File S1.

### 3.4. Pathway Analysis of Differentially Expressed Metabolites Between Low- and High-Motile Semen

To understand how changes in the metabolite levels are related to biological processes and functions, we mapped the DEMs into KEGG pathways (Table 3).

Seven metabolic pathways, including pentose phosphate pathway; terpenoid backbone biosynthesis; and metabolisms related to glycine, serine, and threonine; glycerolipid; glyoxylate and dicarboxylate; purine; and cysteine and methionine, were significantly enriched on Day 0. On Day 7, the number of enriched pathways amounted to 11, with 6 common to Day 0 and 5 additional ones, corresponding to fatty acid biosynthesis and metabolisms related to pyrimidine, glycerophospholipid, nicotinate and nicotinamide, and arginine and proline.

## 4. Discussion

Currently, the chilling of semen samples represents the gold standard for boar ejaculate preservation and subsequent AI. Approximately 99% of porcine AI is realized with freshly extended or preserved semen at 15–17 °C [27]. However, even with the optimization of extenders, only a fraction of the original sample survives preservation. The temperature fluctuations coupled with other intrinsic or extrinsic factors impose an irreversible decline in the percentage of sperm that maintains ultrastructure and biochemical components and, ultimately, sperm functionalities. Specifically, the motility, plasma membrane integrity, and mitochondrial function are compromised due to the excessive generation of reactive oxygen species [2,28]. This process is amplified as the preservation time is lengthened. Interestingly, sperm motility has been positively correlated with the farrowing rate in swine [29,30], and therefore, it represents a core attribute for the success of insemination programs. Moreover, research suggests sperm metabolites may directly or indirectly regulate signaling pathways involved in motility, hyperactivation, and energy metabolism [31]. With growing evidence that ejaculates satisfying industry standards to be processed in AI doses depict differential profiles during storage, allowing us to distinguish ejaculates with good or poor preservability [9,11,32], we investigated the metabolomic profiles of low- and high-motile semen characterized following 7-day storage at 17 °C and identified putative biomarkers. Biomarkers represent an essential toolkit for the early discrimination of semen samples and their optimal usage in AI programs [21,33].

The results of the present study demonstrated a significant decline in semen quality—specifically TM, PM, and NM—in the LM group during storage. In contrast, semen quality in the HM group remained stable, highlighting the superior resilience of these ejaculates. This stability may be attributed to a higher abundance of saturated lipids and cholesterol in the sperm membranes, which enhances membrane integrity and supports motility retention during storage [34]. This observation is consistent with previous reports showing that some boar semen samples stored for 7 days can achieve fertility outcomes comparable with those of fresh semen [35]. Furthermore, ejaculate-specific differences in lipid saturation levels, cholesterol-to-phospholipid ratios, and boar-specific seminal plasma proteins that interact with the sperm membrane may contribute to the observed divergence between LM and HM samples.

The primary class of metabolites were lipids and lipid-like molecules, consistent with previous findings in swine [25,36]. In contrast, it was reported that organic acids and derivatives were the predominant class in bovine [37]. Species-specific differences along with management practices may explain this discrepancy in the dominant class of metabolic products. The classification of the samples based on their respective metabolomic profiles using OPLS-DA resulted in a separation of samples regardless of the storage period and ion modes, indicating that metabolomic data could reflect the difference in motility between the sample groups. However, it is also noteworthy that the variance explained by the OPLS-DA model suggests moderate separation, which may warrant further investigation, especially the functional validation in large-scale studies. Interestingly, only a few DEMs driving the separation of the samples were shared between freshly extended and 7-day stored semen, highlighting the dynamics of the metabolism and associated biological processes during storage. It is well known that spermatozoa are flexible cells, adjusting their metabolism according to substrate availability during storage [38] Furthermore, Mfuzz clustering demonstrated varied metabolomic patterns across sample groups (HM and LM) and storage durations (Days 0 and 7), confirming the dissimilar metabolic profile between HM and LM semen doses and the temporal shift in metabolic activity.

Metabolites play critical roles in the quality of liquid and frozen semen [39,40,41]. In the present study, 43 DEMs were detected between groups of freshly extended semen. Some of these DEMs, including carvone, myricanone, and phosphoserine, known for their potent antioxidant activity, as well as lysoPC(20:3), lysoPC(16:0), lysoPC(18:0), and 1-O-hexadecyl-sn-glycero-3-phosphocholine, recognized for their role as membrane stabilizers, were upregulated in the high-motile semen groups. The positive effects of these DEMs on cells may contribute to the semen’s ability to maintain sperm motility over a longer storage period without significant decreases. A previous study on the metabolomic profiles of sperm with varying abilities to withstand chilled storage reported substantial differences in the expression of antioxidant-related metabolites [36]. Similarly, a study on the metabolomics of freeze-resistant boar sperm emphasized the relevance of antioxidant metabolites [25]. On the other hand, membrane stabilizers such as lysophospholipids might monitor lipid peroxidation, a process whereby reactive oxygen species attack membrane lipids, leading to a loss of motility and viability, morphological and DNA damage, and reduced fertility [3,42]. Interestingly, lysophospholipids have been positively correlated with sperm motility in human sperm cells [43], reinforcing the relevance of lipid metabolites in maintaining sperm quality.

Glyceric acid and lysoPC(20:3) were the shared DEMs across storage with a substantial variation in fold change between Day 0 and Day 7 and were considered as candidate biomarkers for distinguishing high- and low-motile semen. Glyceric acid has been proven to be efficient against cyclophosphamide-induced testicular damage in rats, improving histological characteristics and sex hormones and limiting the lipid peroxidation level [44], indicating its antioxidant potential. Furthermore, glyceric acid is biochemically related to glycolysis through its connection to glyceraldehyde and glycerol metabolism, suggesting a supportive role of glyceric acid in energy substrate availability and, ultimately, in sperm motility. In freshly extended samples, glyceric acid was highly expressed in LM samples, whereas in 7-day-stored samples it was highly expressed in HM samples with a significantly higher fold change. The high expression of glyceric acid in freshly extended LM samples could indicate an intrinsic state of energy and antioxidant demand, depleting cellular glyceric acid content along with the storage, contrary to the case in HM samples. LysoPC(20:3) has been identified in human spermatozoa and, along with other lysophosphatidylcholines (lysoPCs), is involved in maintaining membrane fluidity and curvature, which are critical for sperm motility [43]. During storage, the sperm membrane undergoes oxidative and structural stress. LysoPC(20:3), with its polyunsaturated fatty acid tail, may help preserve membrane dynamics and prevent rigidity, therefore ensuring superior motility of the HM samples. In addition, lysoPCs can act as bioactive lipids, influencing intracellular signaling pathways that regulate ATP production and flagellar movement through calcium channel modulation, which is essential for motility regulation [45]. Likewise, lysoPCs have been shown to modulate oxidative stress, a major factor in sperm quality decline during storage. By modulating reactive oxygen species, lysoPC(20:3) might help preserve mitochondrial function, which is vital for motility. Considering the observed expression patterns for glyceric acid and lysoPC(20:3), it would be beneficial to consider conducting a large-scale study to assess the potential advantages of incorporating these compounds into preservation media.

In the present study, the DEMs were mainly enriched in metabolic pathways of amino acids (glycine, serine, threonine, cysteine, arginine, proline, and methionine), pentose phosphate pathway, glycerolipid metabolism, glyoxylate and dicarboxylate metabolism, and terpenoid backbone biosynthesis. These pathways have been reported in previous studies exploring the metabolomic signatures of boar spermatozoa and seminal plasma with differential abilities to sustain liquid preservation [24,36] or cryopreservation [25,28] and might therefore participate in the regulation of sperm quality. The sperm-derived DEMs might regulate metabolomic pathways to enhance the motility of porcine sperm during preservation. Notably, improved cryotolerance of boar spermatozoa has been related to the increased phosphorylation of serine residues of HSP70 during holding time at 17 °C [46]. Proline is considered a useful reagent for boar sperm cryopreservation as it reportedly enhances semen quality after freezing and thawing [41]. The protective effects of cysteine during storage on sperm parameters, such as motility, have been documented in rams [47,48], bulls [49], and boars [39,50]. Moreover, cysteine enhanced porcine preimplantation embryo development following intracytoplasmic sperm injection [51]. In parallel, the pentose phosphate pathway has been documented to be a major energy metabolic pathway in the maintenance of goat sperm quality during storage [52]. Hence, modulating these pathways might offer novel horizons to prevent sperm-storage-associated damage.

## 5. Conclusions

This study demonstrated the metabolomic differences in boar semen with varying capacities for liquid preservation. It identified key metabolites—primarily involved in amino acid metabolism and the pentose phosphate pathway—that may serve as biomarkers for preservation potential, particularly glyceric acid and lysoPC(20:3). Furthermore, the dynamic nature of the sperm metabolome underscores the metabolic flexibility of sperm in maintaining quality during storage.

## Figures and Tables

**Table 1:** Classification of semen doses based on total sperm motility percentages after chilled storage. Motility and morphology characteristics of individual boar semen doses (*n* = 26; one dose per boar) were assessed both at fresh collection (Day 0) and after chilled storage (Day 7). The classification status for semen was not performed after each replicate (n.d.: not determined).

Replicates	Semen Doses		Day 0 (mean ± sem; %)		Day 7 (mean ± sem; %)	
	*n*	Status	Motility	Morphology	Motility	Morphology
I	4	n.d.	78.53 ± 4.07	86.8 ±3.53	70.08 ± 8.84	83.78 ± 1.44
II	4	n.d.	81.88 ± 2.04	91.25 ± 2.20	74.55 ± 2.47	84.95 ±2.33
III	4	n.d.	84.03 ± 2.35	90.73 ± 0.71	74.28 ±6.60	87.70 ± 4.96
IV	4	n.d.	78.18 ± 2.42	86.88 ± 3.46	75.58 ±2.53	85.08 ±1.93
V	5	n.d.	74.42 ± 4.86	81.42 ± 3.83	71.06 ±2.62	73.80 ± 2.39
VI	5	n.d.	78.53 ± 1.07	86.24 ± 1.72	76.22 ± 2.11	83.78 ± 1.44
Total	26		79.26 ± 1.36	87.22 ± 1.45	73.63 ± 1.02	83.18 ± 1.97
Semen Classification	6	LM	79.92 ± 2.18	88.97 ± 1.41	59.40 ± 3.35	79.93 ± 2.61
	14	MM	73.84 ± 2.15	83.44 ± 2.03	77.75 ± 0.82	80.16 ± 1.53
	6	HM	84.02 ± 1.84	89.25 ± 1.17	83.73 ± 1.17	89.45 ± 2.30

**Table 2:** KEGG variable importance in projection (VIP) scores of the top metabolites driving sample separation. The VIP scores, derived from OPLS-DA analysis, were used to identify the top metabolites that differentiate low-motile (LM) and high-motile (HM) boar semen in positive (ESI+) and negative (ESI−) ion modes on Day 0 and Day 7.

HMDB_ID	Compound Names	FC (HM/LM)	*t*-Test	VIP	ESI	Day
HMDB0059875	Carvotanacetone	1.8373	0.0009	2.2	+	0
HMDB0255129	N-Dodecylsarcosinate	15.5784	0.0010	2.4	−	0
HMDB0251781	Emulphor	2.3938	0.0043	2.0	+	0
HMDB0037012	2-Hydroxy-p-mentha-1,8-dien-6-one	1.2757	0.0053	2.0	+	0
HMDB0013648	Palmitoleoylethanolamde	1.3352	0.0064	2.1	+	0
HMDB0001090	5-Diphosphomevalonic acid	2.3281	0.0092	2.4	−	0
HMDB0000139	Glyceric acid	0.5340	0.0102	2.1	−	0
HMDB0248559	Arachidoyl ethanolamide	2.0082	0.0126	2.2	+	0
HMDB0341228	4-Indolecarbaldehyde	2.0330	0.0152	2.1	−	0
HMDB0029104	Tyrosyl-glutamate	0.6406	0.0168	2.0	+	0
HMDB0243890	1-O-Hexadecyl-sn-glycero-3-phosphocholine	2.5811	0.0169	2.0	−	0
HMDB0254257	Lykurim	0.5465	0.0229	2.1	+	0
HMDB0116636	PG(a-13:0/a-13:0)	9.4155	0.00003	2.1	−	7
HMDB0028831	Glutamyltyrosine	7.9348	0.00003	2.1	−	7
HMDB0011517	LysoPE(20:4)	8.1406	0.00008	2.1	−	7
HMDB0251781	Emulphor	2.2117	0.00010	2.5	+	7
HMDB0062549	2-Hydroxystearic acid	26.8913	0.00014	2.2	−	7
HMDB0041249	6-Hydroxyshogaol	4.4944	0.00020	2.5	+	7
HMDB0248559	Arachidoyl ethanolamide	1.9631	0.00067	2.3	+	7
HMDB0010404	LysoPC(22:6)	5.2845	0.00119	2.1	−	7
HMDB0000806	Myristic acid	5.0312	0.00250	2.1	−	7
HMDB0010393	LysoPC(20:3)	5.3619	0.00407	2.1	−	7
HMDB0000824	Propionylcarnitine	0.5699	0.00598	2.0	+	7
HMDB0000791	Octanoylcarnitine	0.4283	0.00729	2.1	+	7
HMDB0266619	PA(8:0/PGJ2)	3.1500	0.00888	2.0	+	7
HMDB0266633	PA(8:0/20:4+=O(5))	3.3804	0.00995	2.1	+	7
HMDB0001406	Niacinamide	0.4928	0.0115	2.2	+	7
HMDB0000651	Decanoylcarnitine	0.5544	0.0138	2.1	+	7
HMDB0015353	Budesonide	106.9614	0.0269	2.0	−	7
HMDB0032575	Butylparaben	0.2144	0.0296	2.1	+	7
HMDB0029817	Ethyl salicylate	0.2226	0.0304	2.0	+	7
HMDB0034240	Styrene	2.4391	0.0304	2.0	+	7
HMDB0255266	N,N-Dimethyloctadecylamine	0.0204	0.0413	2.1	+	7
HMDB0002833	Testosterone sulfate	8.8909	0.0453	2.0	−	7
HMDB0030631	Rubraflavone D	2.6503	0.0491	2.0	+	7

**Table 3:** KEGG pathways of dysregulated metabolites between high- and low-motile boar semen on Days 0 and 7 of storage. Data were acquired in positive (ESI+) and negative (ESI−) ion modes.

Storage Duration	Pathways	*p*-Value	Modes
Day 0	Glycine, serine, and threonine metabolism	5.525 × 10^−4^	ESI−
	Pentose phosphate pathway	3.117 × 10^−3^	ESI−
	Glycerolipid metabolism	3.117 × 10^−3^	ESI−
	Glyoxylate and dicarboxylate metabolism	3.117 × 10^−3^	ESI−
	Terpenoid backbone biosynthesis	3.340 × 10^−3^	ESI−
	Cysteine and methionine metabolism	1.639 × 10^−2^	ESI−
	Glycerolipid metabolism	2.213 × 10^−2^	ESI+
	Purine metabolism	6.147 × 10^−2^	ESI+
Day 7	Fatty acid biosynthesis	7.849 × 10^−4^	ESI−
	Pentose phosphate pathway	1.259 × 10^−3^	ESI−
	Glycine, serine, and threonine metabolism	1.259 × 10^−3^	ESI−
	Glycerolipid metabolism	1.259 × 10^−3^	ESI−
	Glyoxylate and dicarboxylate metabolism	1.259 × 10^−3^	ESI−
	Pyrimidine metabolism	1.289 × 10^−3^	ESI−
	Purine metabolism	2.214 × 10^−3^	ESI−
	Glycerophospholipid metabolism	2.395 × 10^−3^	ESI−
	Terpenoid backbone biosynthesis	4.938 × 10^−3^	ESI−
	Glycerolipid metabolism	8.543 × 10^−3^	ESI+
	Glycerophospholipid metabolism	8.543 × 10^−3^	ESI+
	Nicotinate and nicotinamide metabolism	9.484 × 10^−3^	ESI+
	Arginine and proline metabolism	1.695 × 10^−2^	ESI+

## Data Availability

The data used in the study are available within the manuscript and in the Supplementary File.

## Supplementary Materials

The following supporting information can be downloaded at https://www.mdpi.com/article/10.3390/ani15152163/s1: Supplementary File S1: Total and differentially expressed metabolites of high and low motile Duroc boar spermatozoa following chilled preservation.

## Abbreviations

The following abbreviations are used in this manuscript:

AI	Artificial insemination
DEMs	Differentially expressed metabolites
HM	High motile
LM	Low motile
MM	Medium motile
MOT	Total motility
PM	Progressive motility
